# Effect of the prebiotic fiber inulin on cholesterol metabolism in wildtype mice

**DOI:** 10.1038/s41598-018-31698-7

**Published:** 2018-09-05

**Authors:** Rima H. Mistry, Fangjie Gu, Henk A. Schols, Henkjan J. Verkade, Uwe J. F. Tietge

**Affiliations:** 1 Department of Pediatrics, University of Groningen, University Medical Center Groningen, Groningen, The Netherlands; 2 Laboratory of Food Chemistry, Wageningen University and Research, Wageningen, The Netherlands

## Abstract

Dietary non-digestible carbohydrates are perceived to improve health via gut microbiota-dependent generation of products such as short-chain fatty acids (SCFA). In addition, SCFA are also precursors for lipid and cholesterol synthesis potentially resulting in unwanted effects on lipid metabolism. Inulin is a widely used model prebiotic dietary fiber. Inconsistent reports on the effects of inulin on cholesterol homeostasis have emerged in humans and preclinical models. To clarify this issue, the present study aimed to provide an in-depth characterization of the effects of short-chain (sc)- and long-chain (lc)- inulin on cholesterol synthesis, absorption and elimination in mice. Feeding wildtype C57BL/6J mice diets supplemented with 10% (w/w) of either sc- or lc-inulin for two weeks resulted in approximately 2.5-fold higher fecal SCFA levels (P < 0.01) compared with controls, but had no significant effects on plasma and liver lipids. Subtle shifts in fecal and plasma bile acid species were detected with beta-muricholic acid increasing significantly in plasma of the inulin fed groups (1.7-fold, P < 0.05). However, neither sc-inulin nor lc-inulin affected intestinal cholesterol absorption, mass fecal cholesterol excretion or trans-intestinal cholesterol excretion (TICE). Combined, our data demonstrate that sc- and lc-inulin have no adverse effects on cholesterol metabolism in mice despite increased generation of SCFA.

## Introduction

Inulin is a soluble non-digestible carbohydrate studied for its health benefits such as improved bowel movements, lowering blood glucose levels and potential lipid modulating effects^1–4^. Inulin is naturally available in many types of plants. As ingredient for the food industry native inulin is mainly extracted from chicory roots^5^. Structurally, inulin is composed of β-2,1-linked fructans with a terminal glucose. The chain length varies, in other words, there is a varying degree of polymerization (DP), which on average ranges between 2–60^6,7^. The β-2,1-linked fructose units present in all types of inulin prevent it from digestion by mammalian hydrolytic enzymes in the intestine^8^.

As a result, inulin undergoes fermentation by the microbiota in different parts of the intestine resulting in the production of short-chain fatty acids (SCFA)^9^. Recently, several studies showed that SCFA could potentially regulate metabolic pathways in the liver as well as other organs and thereby exert physiological health effects^10–12^. However, gut-derived SCFA such as acetate, for example, can potentially serve as a precursor for cholesterol and fatty acid synthesis^13^. Thereby, inulin might exert adverse effects on metabolism by increasing plasma cholesterol levels and thus the risk for atherosclerotic cardiovascular disease. Previous studies in animal models as well as human trials have been ambiguous in terms of cholesterol modulating properties of inulin. Oligofructose and inulin have been shown to lower plasma cholesterol in apolipoprotein E-deficient mice^14^. However, the effects were not evident in germ-free rats inoculated with human fecal microflora nor in obese Zucker rats^15,16^. While in healthy humans cholesterol levels remained unchanged with inulin consumption, dyslipidemic patients consuming inulin for a longer duration had lower cholesterol levels^17–20^.

In the present study we aimed to elucidate in detail the effects of inulin on cholesterol absorption and elimination. We performed an *in vivo* dietary intervention study in mice using either short-chain enriched (sc-) or long-chain (lc-) enriched inulin. Our data demonstrate that, despite a substantially increased intestinal production of SCFA, inulin has no adverse effects on *de novo* cholesterol synthesis, intestinal cholesterol absorption or cholesterol elimination pathways.

## Results

### Sc-and lc- inulin increase the intestinal production of SCFA

**Figure 1:**
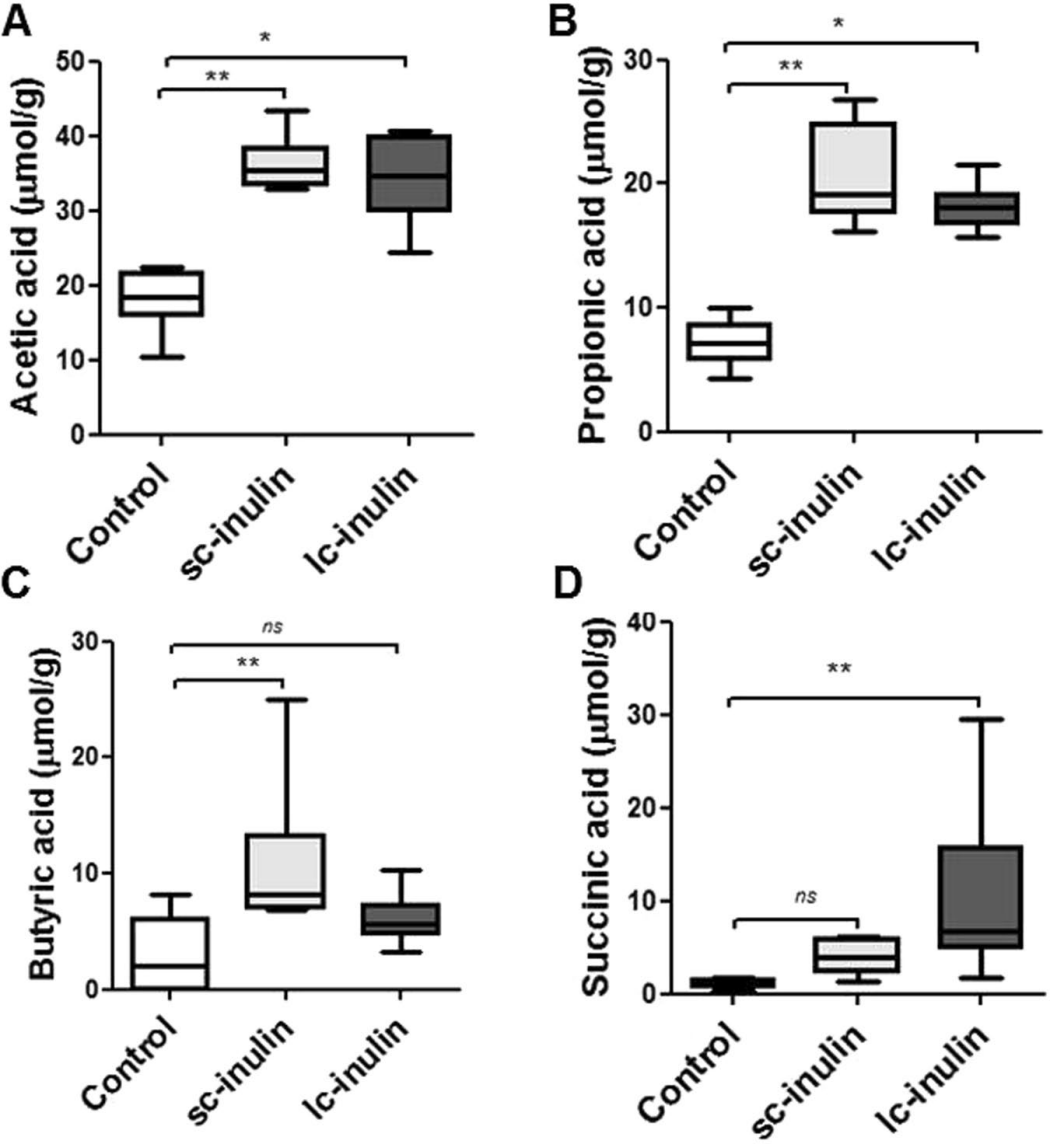
Sc- and lc- inulin increase fecal short-chain fatty acid content. (**A**) Acetic acid; (**B**) propionic acid; (**C**) butyric acid; (**D**) succinic acid. Following two weeks administration of the respective diets, feces were collected over a period of 24 h. Fecal samples from each mouse were used for extraction and analysis of SCFA. Data are presented as box plots showing median (interquartile range) and min/max; at least n = 6 for each group; ns, not significant. Statistically significant differences are indicated as *P < 0.05; **P < 0.01.

Two weeks of dietary intervention with sc- or lc-inulin did not change body weight compared to mice fed control diet (data not shown). Sc-inulin significantly increased fecal acetic (2-fold), propionic (2-fold) and butyric acid (4-fold) levels (each P < 0.01, Fig. 1A,B and C). On the other hand, lc-inulin increased acetic (2-fold, P < 0.05), propionic (2-fold, P < 0.05) and succinic acid (9-fold, P < 0.01) production (Fig. 1A,B and D), while butyric acid production was comparable to the control group. Overall, considerable shifts were observed in terms of the ratio (Ac:Pr:Bu:Su) of SCFA generated in sc-inulin (51:29:15:6) and lc-inulin (50:26:9:15) fed groups compared to mice fed control diet (61:25:10:4).

### Impact of sc- and lc- inulin on cholesterol metabolism

**Figure 2:**
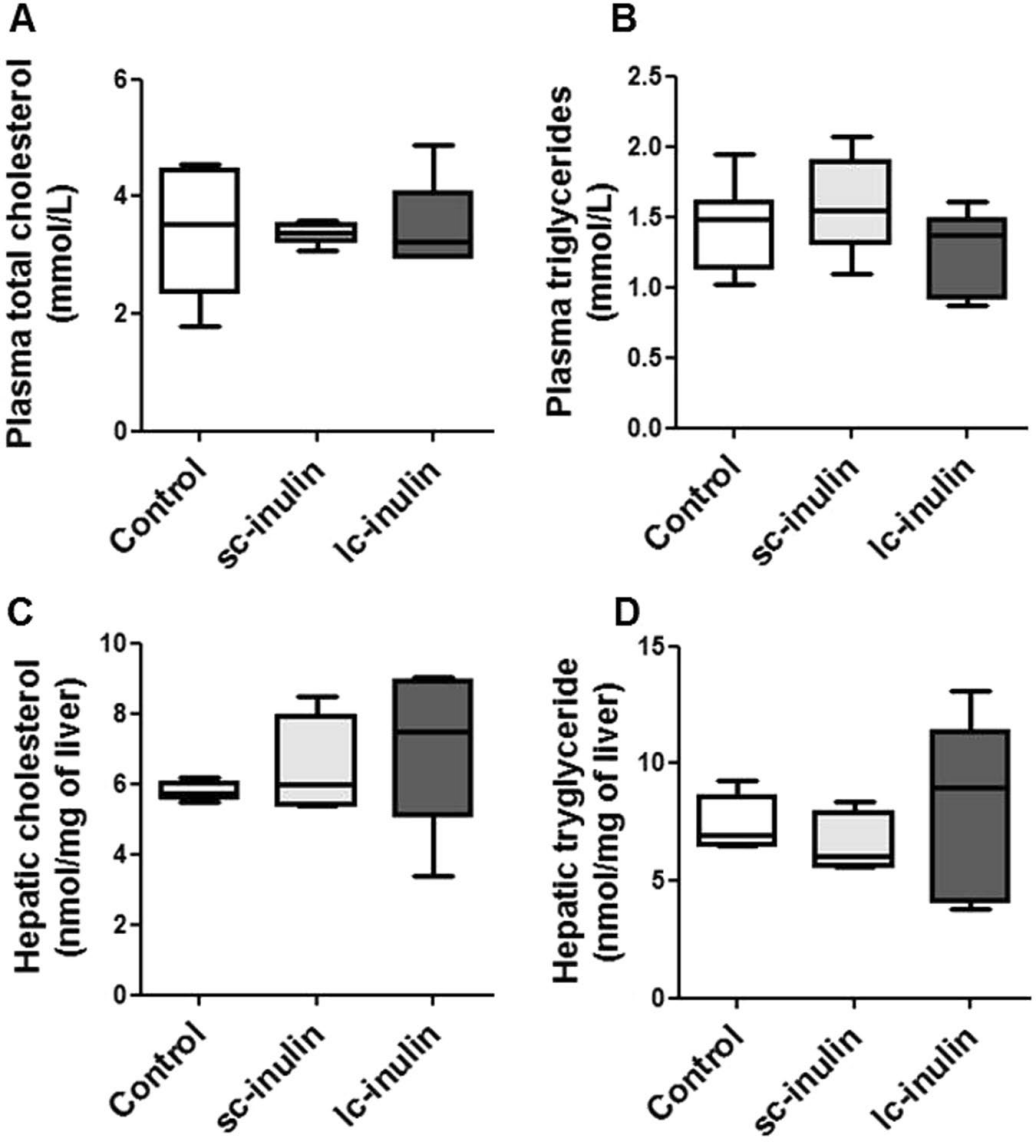
Sc- and lc- inulin have no effect on plasma and hepatic lipid levels. (**A**) Plasma total cholesterol (TC); (**B**) plasma triglycerides (TG); (**C**) hepatic TC and (**D**) hepatic TG. At the time of sacrifice plasma samples were collected from the experimental mice for total cholesterol and triglyceride measurements. Livers were excised, weighed and stored at −80 °C for later lipid analysis. Data are presented as box plots showing median (interquartile range) and min/max; at least n = 6 for each group.

**Figure 3:**
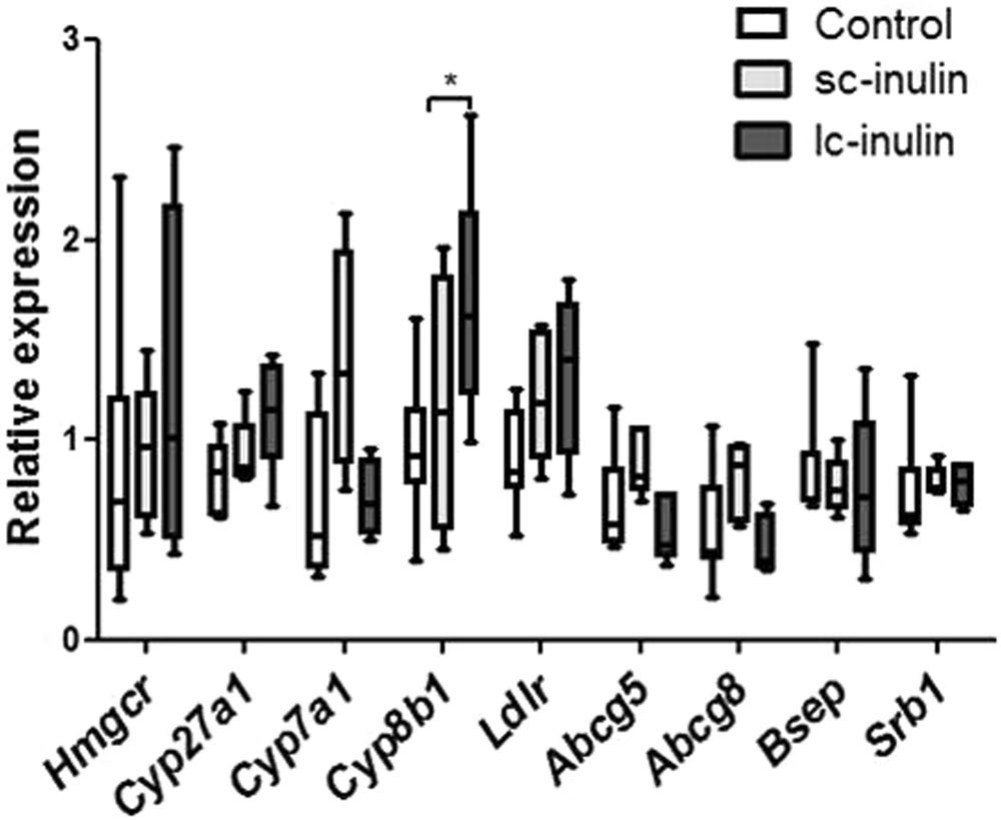
Hepatic gene expression in mice fed sc- and lc-inulin. At day 14 livers were excised and stored at −80 °C for later analysis. Quantitative real-time PCR was carried out as described in methods. Individual genes are expressed as a ratio to the expression of the housekeeping gene cyclophilin. Data are presented as box plots showing median (interquartile range) and min/max; at least n = 6 for each group. Statistically significant differences are indicated as *P < 0.05.

At the end of the dietary intervention, plasma cholesterol and triglyceride levels were not different among the experimental groups (Fig. 2A,B), as were hepatic cholesterol and triglyceride contents (Fig. 2C,D). Hepatic mRNA expression of cholesterol synthesis-related genes such as *Hmgcr* remained unchanged in all groups (Fig. 3).

**Figure 4:**
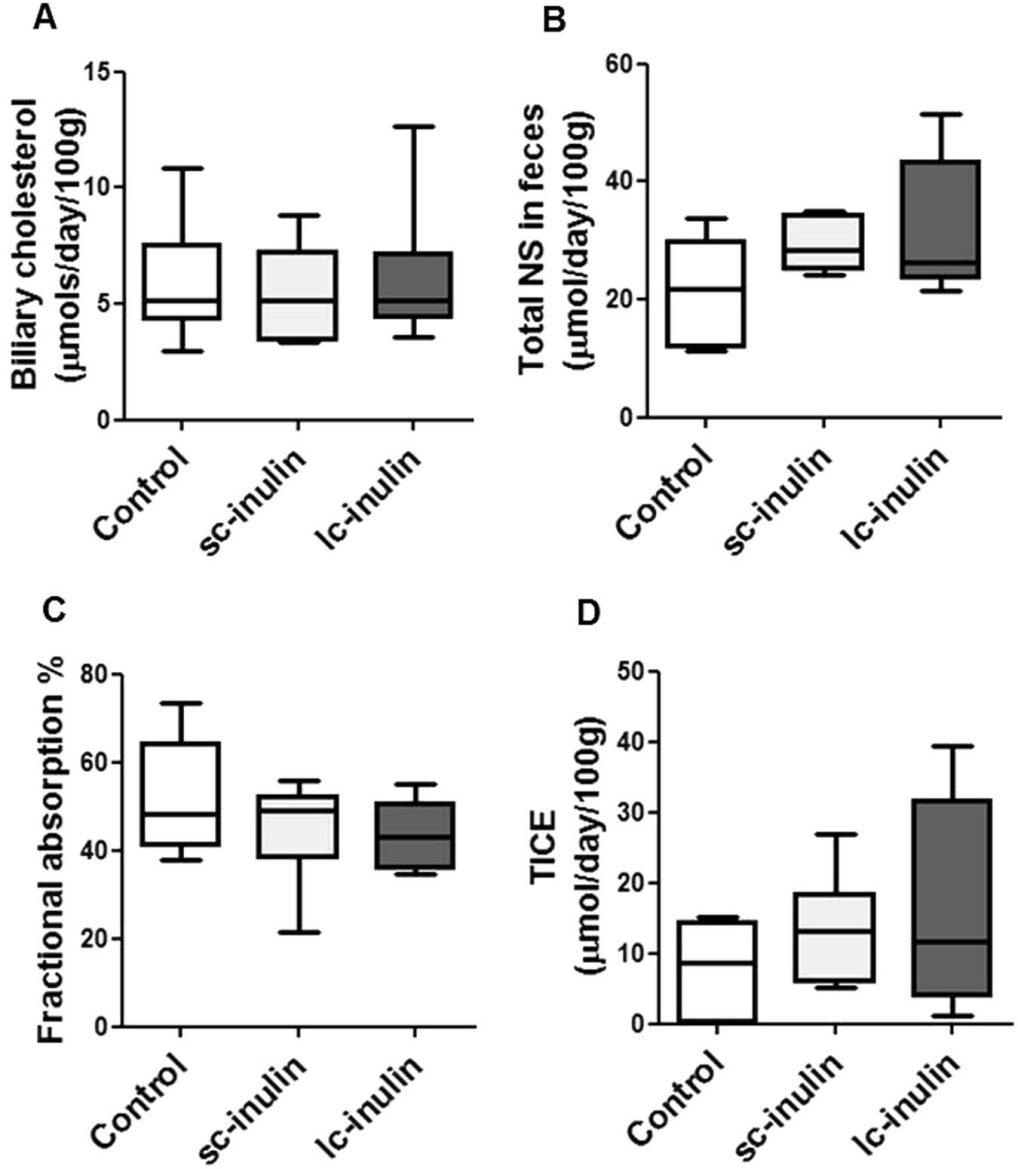
Cholesterol excretion pathways remain unaffected by dietary sc- and lc-inulin. (**A**) Biliary cholesterol secretion; (**B**) total fecal neutral sterol (NS) excretion; (**C**) intestinal fractional cholesterol absorption; (**D**) trans-intestinal cholesterol efflux (TICE). At day 14 bile cannulation was performed for continuous collection of bile. Feces were collected over 72 h and analyzed for NS as detailed in methods. Fractional cholesterol absorption was measured using the plasma dual isotope method. Data are presented as box plots showing median (interquartile range) and min/max; at least n = 6 for each group.

Neither sc- nor lc-inulin had significant effects on biliary cholesterol secretion and fecal neutral sterol excretion (Fig. 4A,B). Fractional cholesterol absorption upon sc- or lc-inulin feeding was similar to that in controls (Fig. 4C). Based on these data we also calculated excretion of cholesterol through the TICE pathway. The data show that in inulin fed mice TICE remained comparable to controls (Fig. 4D).

### Sc- and lc-inulin induce subtle shifts in biliary and fecal bile acid composition

**Figure 5:**
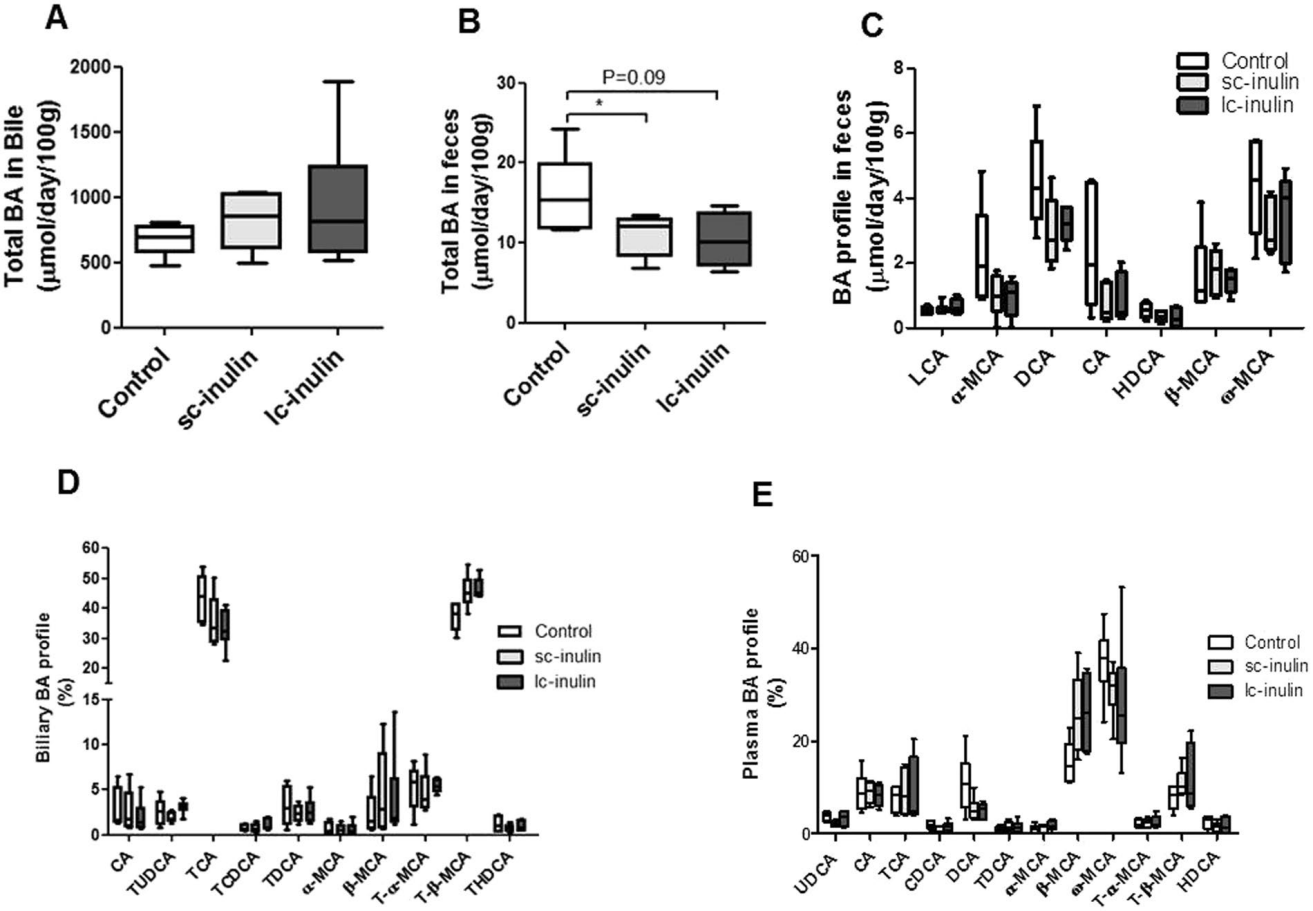
Sc- and lc-inulin induce subtle shifts in bile acid profiles. (**A**) Total biliary BA secretion; (**B**) total fecal BA excretion; (**C**) BA profile in feces; (**D**) Biliary BA profile and; (**E**) Plasma BA profile. At day 14 plasma, fecal and bile samples were collected and processed for bile acid analysis as detailed in methods. Data are presented as box plots showing median (interquartile range) and min/max; at least n = 6 for each group. Statistically significant differences are indicated as *P < 0.05, **P < 0.01. Abbreviations: ω-MCA, ω-muricholic acid; β-MCA, β-muricholic acid; α-MCA, α-muricholic acid; CA, cholic acid; CDCA, chenodeoxy-cholic acid; LCA, lithocholic acid, DCA, deoxycholic acid; UDCA, ursodeoxy-cholic acid; HDCA, hyodeoxycholic acid; T-BA, taurine-conjugated bile acids.

Prebiotics can impact the composition of the intestinal microbiota potentially resulting in altered bile acid profiles^21^. Our analyses indicate that total bile acids in bile remained comparable in all groups (Fig. 5A), while total bile acids were significantly lower in feces of sc-inulin fed mice (1.5-fold compared to control P < 0.05, Fig. 5B), while only a trend was observed for lc-inulin (P = 0.09, Fig. 5B). Correspondingly, a trend towards a decrease in some fecal bile acid species was noted in sc-inulin fed mice such as for deoxycholic acid (DCA, P = 0.07, Fig. 5C). Composition of bile acids in bile remained unaltered with sc- and lc-inulin feeding (Fig. 5D). With respect to plasma bile acids (Fig. 5E) sc-inulin fed mice showed a decreasing trend in the levels of chenodeoxycholic (CDCA, P = 0.06), deoxycholic acid (DCA, P = 0.06) and higher level of β-muricholic acid (β-MCA, P = 0.08, Fig. 5E). Lc-inulin fed mice tended to have lower levels of plasma deoxycholic acid (DCA, P = 0.05) and higher levels β-muricholic acid (β-MCA, P = 0.06, Fig. 5E). Hepatic mRNA gene expression of *Cyp8b1*, which is of major importance for the composition of the bile acid pool, was significantly upregulated by the lc-inulin diet (P < 0.01, Fig. 3), which, however, did not exert major effects on the bile acid pool as described above. On the other hand, *Cyp7A1* remained largely unaffected.

## Discussion

In the present study we aimed to investigate the effects of inulin on cholesterol homeostasis. We demonstrate that despite of a substantial increase and alterations in the ratio of microbiota-derived SCFA by sc-inulin and lc-inulin this did not translate into adverse effects on cholesterol metabolism. Such an assumption appeared plausible and thus worth investigating, since it had been shown that gut-derived acetic and butyric acid are incorporated into the cholesterol synthesis pathway in the liver^13^. Acetic acid is converted into acetyl-coA, a precursor in the hepatic cholesterol synthesis pathway^22^. Butyric acid is involved in mitochondrial fatty acid oxidation eventually also yielding acetyl-coA^23^. In our study, we detected increased levels of fecal acetic acid and butyric acid particularly with sc-inulin. However, cholesterol *de novo* synthesis remained unchanged as indicated by the comparable levels of hepatic *Hmgcr* mRNA expression among the groups and also by the fact that hepatic cholesterol concentrations remained unchanged. Propionic acid on the other hand, can attenuate lipid biosynthesis in liver^24^. Elevated propionic acid in mice fed either sc- or lc- inulin, however, did not coincide with altered lipid levels in plasma or liver or changes in the mRNA expression of relevant genes for cholesterol metabolism.

In general, with respect to plasma and liver, intestinal fermentation of fibers has been shown to suppress plasma and liver cholesterol as well as triglyceride levels^25^. Here specifically the cecum has been shown to be an anatomically relevant location for these metabolic effects by elegant surgical intervention studies^26^. These observations are presumably due to SCFA or other as of yet less well characterized bacterial products^10,27^. For example, addition of propionate to hepatocytes *in vitro* was indicated to decrease cholesterol synthesis by reducing HMGCoAR activity^28,29^. The mechanism of this result has not been clearly elucidated; however, it could be speculated that the AMPK pathway is involved, which is on the one hand known to be activated by SCFA and on the other to inhibit HMGCoAR activity^28,30^. Further, also via AMPK activation, hepatic fatty acid oxidation is increased by SCFA, while fatty acid synthesis is lower^10^. These effects are thought to result in net improvements in hepatic triglyceride storage. The underlying molecular mechanisms, however, are still incompletely understood.

The present data also indicate that sc- and lc-inulin, despite profoundly increasing SCFA levels, did not affect the metabolically relevant cholesterol fluxes in the intestine. First, intestinal cholesterol absorption studies revealed no significant changes in inulin-fed animals compared to controls. Second, cholesterol excretion *via* the feces remained unaffected as well. As a consequence, intestinal excretion of cholesterol *via* the TICE pathway remained unaltered by the experimental diet.

Cholesterol can be eliminated from the body as fecal neutral sterols and as fecal bile acids. Bile acids are produced as a result of metabolic conversion of cholesterol in the liver and are secreted into the intestine *via* the biliary pathway. We also analyzed the effects of dietary inulin on biliary secretion as well as on fecal excretion of bile acids. The total biliary bile acid secretion tended to increase, but significance was not reached. Fecal excretion of bile acids exhibited a decreasing trend potentially reflecting increased absorption of bile acids. In addition, subtle changes in the bile acid profiles were detected in plasma as well as in feces, suggesting a potential increase in absorption of certain bile acids species. We believe, however, that these changes are not of major physiological importance.

Microbial activity is crucial for modification of bile acid species by deconjugation and hydroxylation of bile acids which eventually results in the formation of secondary bile acids^31^. No significant alterations in fecal secondary bile acid levels occurred indicating that at least the microbial composition with respect to bile acid metabolism remained unaltered upon inulin feeding. Combined, our results indicate that neither sc- nor lc-inulin feeding in wildtype mice adversely affects relevant parameters of cholesterol metabolism.

## Materials and Methods

### Experimental animals

Nine weeks old conventional male C57BL/6 mice were obtained from Harlan (Horst, The Netherlands) and were housed in a temperature controlled room with alternating 12 h light-dark cycles. All animal experiments were approved by the Committee of Animal Experimentation at the University of Groningen and performed in accordance with the Dutch national Law on Animal Experimentation (Wod) as well as international guidelines on animal experimentation. Three different diets were randomly assigned to mice and provided *ad libitum* for a period of 14 days in a parallel design. Baseline diet (supplied by Safe Diets, Augy, France) contained 60.94% corn starch, 0.06% cholesterol, 20% caseinate, 0.3% L-cystine, 7% carbohydrate mix (sucrose:maltodextrin, 50:50), 7% soya bean oil, 0.2% choline bitartrate, 3.5% mineral mixture (AIN 93 M/G), 1% vitamin mixture (w/w, AIN 93 M/G). A modified diet contained 10% lc-inulin (Frutafit® TEX!, DP of 10–60, Sensus, The Netherlands) or sc-inulin (Frutafit® CLR, DP of 2–40, Sensus, The Netherlands) replacing an equal amount of corn starch (50.94% corn starch, 0.06% cholesterol, 20% caseinate, 0.3% L-cystine, 7% carbohydrate mix, 7% soya bean oil, 0.2% choline bitartrate, 3.5% mineral mixture, 1% vitamin mixture).

### Bile collection and analysis of bile acid, cholesterol and phospholipid excretion

The gallbladder was cannulated under anaesthesia (hypnorm 1 ml/kg body weight; diazepam 10 mg/kg body weight). Bile was collected for 20 minutes and the secretion rates were determined gravimetrically. Lipid extraction from bile was performed according to the general procedure of Bligh and Dyer with minor modifications^32^. Cholesterol was measured with colorimetric assays (Roche Diagnostic, Basel, Switzerland). Phospholipids were determined by measuring phosphate as described, bile acid profiles were generated using liquid chromatography tandem MS (LC-MS/MS) as published previously^33^. Secretion of bile acids, cholesterol and phospholipids was calculated by measuring the respective concentrations and multiplying with bile secretion rates.

### Plasma lipid and lipoprotein analysis

At the time of sacrifice blood was collected by heart puncture using EDTA as anti-coagulant. Plasma was obtained and aliquots were stored at −80 °C until further analysis. Commercially available colorimetric assays were used to measure plasma total cholesterol and triglycerides (Roche Diagnostic, Basel, Switzerland). Plasma bile acid concentrations in each mouse were measured using LC-MS/MS as described^33^.

### Analysis of hepatic lipid composition

At the time of sacrifice the liver was collected and homogenized in PBS. Lipids were extracted from the homogenates according to the general procedure of Bligh and Dyer with minor modifications and redissolved in water containing 2% Triton X-100^32^. Total cholesterol and triglyceride content was measured using commercially available kits (Roche Diagnostics).

### Fecal neutral sterols, bile acids and SCFA analysis

Mice were housed individually and fecal samples were collected at the end of the dietary intervention over a period of 24 h. Fecal samples of each mouse were dried, weighed and ground. Neutral sterols and bile acids were extracted and their concentrations were measured using gas liquid chromatography^32^. SCFA (acetic acid, propionic acid and butyric acid) and other organic acids (succinic acid and lactic acid) were quantified based on a method published previously with some modifications^34^. The acids were extracted by mixing thoroughly 50 mg fecal samples into 0.35 ml of 50 mM sulfuric acid and 0.025 ml of 4 mg/ml 2-ethylbutyric acid (internal standard). After centrifugation at 18,600 g and 4 °C for 20 minutes, the supernatant was analyzed by high performance liquid chromatography coupled to a refractive index detector (HPLC-RI) as described in literature^34^.

### Cholesterol absorption studies and calculation of TICE

Fractional cholesterol absorption was measured with an adapted plasma dual isotope ratio method using blood samples obtained after intravenous (D7) and oral (D5) administration of stable isotopically labelled cholesterol as described before^35^. Trans-intestinal cholesterol efflux (TICE) is calculated according to the following formula: TICE = fecal cholesterol excretion − [% intestinal cholesterol absorption × (biliary cholesterol secretion + dietary cholesterol intake)].

### Real-time PCR for hepatic gene expression analysis

Using TriReagent (Sigma) total mRNA was extracted from the liver and quantified with a Nanodrop ND-100UV-vis spectrophotometer (NanoDrop Technologies Wilmington DE). cDNA was synthesized using 1 µg of total RNA and reagents from Invitrogen (Carlsbad CA). Real-time PCR was performed on an ABI Prism 7700 system (Applied Biosystems, Darmstadt Germany). Primers were synthesized by Eurogentec (Seraing, Belgium). For each gene, mRNA expression was calculated relative to the housekeeping gene cyclophilin.

### Statistics

Statistical analysis was performed using GraphPad Prism software (San Diego, CA). All data are presented as median (interquartile range). Statistically significant differences between groups were determined using one-way ANOVA and Turkey test for post-hoc analysis. P-values below 0.05 were considered statistically significant.

## Data Availability

The datasets generated and analyzed during the current study are available from the corresponding author on reasonable request.
